# Neurocognitive Effects of Neurofeedback: A Systematic Review of the Applicability and Therapeutic Effect in Patients with Schizophrenia Spectrum Disorders, Psychosis or Clinical High Risks for Psychosis

**DOI:** 10.1007/s10484-026-09773-x

**Published:** 2026-02-19

**Authors:** Giulia Schilirò, Graziella Chiara Prezzavento, Sergio Triscari, Gabriele Pace, Simona Lanzafame, Pasquale Caponnetto

**Affiliations:** 1 Department of Educational Sciences, Section of Psychology, University of Catania, Catania, 95123 Italy; 2 Psychiatric Unit, Policlinico University Hospital “G. Rodolico- San Marco”, Catania, 95123 Italy; 3 Department of Clinical and Experimental Medicine, University of Catania, Catania, 95123 Italy

**Keywords:** EEG-biofeedback, Neurofeedback, Neurocognitive changes, Humoral changes, Schizophrenia spectrum disorders

## Abstract

Schizophrenia Spectrum Disorders are complex mental health conditions that significantly impact cognitive function and quality of life. While pharmacological and psychotherapeutic interventions are available, their effectiveness remains limited, particularly for negative symptoms and cognitive impairments. These limitations, alongside drug side effects and adherence difficulties, highlight the need for new treatments. Cognitive remediation strategies like Neurofeedback show promise by harnessing neuroplasticity. This systematic review aims to evaluate the neurocognitive and humoral changes induced by Neurofeedback and its therapeutic effects in patients with schizophrenia spectrum disorders. Our review was conducted following PRISMA guidelines. Databases including EMBASE, ScienceDirect, Scopus, PsycINFO, and MEDLINE were searched for relevant studies: 14 studies, 10 RCTs, and 4 Clinical trials were selected. Inclusion criteria encompassed studies involving patients with schizophrenia spectrum disorders, Neurofeedback interventions, and outcomes related to neurocognitive and humoral changes. The Cochrane Risk-of-Bias Tool for randomized trials (RoB 2) was used to assess the quality of included studies. The reviewed studies suggest that Neurofeedback shows promise in addressing various aspects of schizophrenia spectrum disorders. Improvements were observed in processing speed, social functioning, working memory, and emotional regulation. Several studies reported successful modulation of brain activity in regions associated with auditory hallucinations. Neurofeedback training also led to increased functional connectivity between language networks and the default mode network. Some studies found improvements in brain-derived neurotrophic factor (BDNF) levels, self-efficacy, and clinical symptoms in schizophrenia patients. Future research should focus on personalizing Neurofeedback approaches and exploring their mechanisms of action in the context of schizophrenia pathophysiology.

## Introduction

Schizophrenia is a complex spectrum of mental disorders characterized by dissociation in thought, affectivity, and behavior, resulting in a profound fragmentation of mental life. The term “schizophrenia,” coined by Swiss psychiatrist Eugen Bleuler, emphasizes the concept of “splitting of the mind,” shifting the focus from Kraepelin’s earlier classification of “Dementia Praecox,” which highlighted early onset and cognitive decline, high relapse and disability, resulting in 13.4 million years of disability-related life loss (YLDs) (Charlson et al., 2018). Between 1990 and 2019, the prevalence of schizophrenia rose from 14.2 to 23.6 million, incidence increased from 941,000 to 1.3 million (Solmi et al., 2023).

The etiology of the disorder is multifactorial. It involves genetic predispositions, such as mutations in the DISC1 gene, which guides neuronal migration and connectivity during brain development, NRG1, which regulates synaptic plasticity, and COMT, which metabolizes dopamine in the prefrontal cortex, affecting cognitive and emotional regulation, interacting with environmental risk factors including perinatal complications, cannabis use, and exposure to stress (Wahbeh & Avramopoulos, 2021).Neuroimaging studies have revealed structural and functional abnormalities in schizophrenia, including cortical gray matter reduction, prefrontal cortex dysfunction, and dysregulated dopamine transmission. Clinically, the disorder manifests with positive symptoms (e.g., delusions, hallucinations), negative symptoms (e.g., avolition, alogia), and significant cognitive impairments that severely impact quality of life and social functioning (APA, 2022).

Despite the availability of pharmacological and psychotherapeutic interventions, current treatments often show limited efficacy, particularly in addressing negative symptoms, such as apathy, anhedonia, avolition, and reduced emotional expression, and cognitive deficits, including impairments in attention, working memory, and executive functioning (Lutgens et al., 2017). These challenges, coupled with medication side effects and adherence issues, highlight the need for innovative, integrative therapeutic strategies (Leichsenring et al., 2019). Among emerging approaches, cognitive remediation therapies such as Neurofeedback (NFB) are gaining traction for their potential to enhance neuroplasticity and promote behavioral, cognitive and emotional rehabilitation in people with schizophrenia (Li et al., 2024).

Neurofeedback (NFB), is a non-invasive technique aimed at optimizing brain function by training individuals to modulate their brainwave activity (Gruzelier, 2014). This approach involves two core processes: recording the brain’s electrical activity and delivering patients real-time auditory and visual feedback (Chiasson et al., 2023). Through a brain-computer interface, individuals are provided with instant feedback on their EEG, enabling them to consciously regulate specific neural activities and achieve desired states, such as relaxation or heightened focus. NFB operates on the principles of operant conditioning, reinforcing optimal brainwave patterns while suppressing undesired ones (Marzbani et al., 2016).

The primary goal of NFB is not just symptom reduction but fostering durable changes in neural functioning, thereby improving cognitive self-regulation and emotional control (Marzbani et al., 2016). This method has demonstrated efficacy across a range of disorders, including ADHD, PTSD, anxiety, and depression (Askovic et al., 2023). In schizophrenia, its ability to directly target neurophysiological dysfunctions holds promise for mitigating cognitive and emotional deficits (Oprea et al., 2024). Preliminary studies indicate improvements in areas such as brain connectivity, working memory, attention, and emotional regulation. Additionally, the customizable nature of NFB, which adapts to individual neurophysiological profiles, enhances its therapeutic potential (Orndorff-Plunkett et al., 2017).

The integration of EEG-biofeedback into schizophrenia treatment represents a novel approach aimed at addressing the disorder’s complex neurocognitive and emotional impairments. Conventional treatments primarily target symptoms without directly influencing the underlying neurophysiological dysfunctions. NFB offers a complementary strategy by leveraging the brain’s inherent neuroplasticity to rewire dysfunctional circuits and promote adaptive neural activity (Turiaco et al., 2024). For schizophrenia spectrum disorders, this approach is particularly relevant given the central role of brain connectivity deficits in cognitive impairments and negative symptoms(Gandara et al., 2020).

Early evidence suggests that NFB may enhance functional connectivity between key brain networks, such as the default mode network and regions implicated in auditory hallucinations (Zhao et al., 2018). Furthermore, studies report potential improvements in neurocognitive functions, such as memory, language processing, and sustained attention, along with reductions in negative symptoms (Pazooki et al., 2019). These findings highlight NFB’s capacity to address the core challenges of schizophrenia beyond symptom management, potentially improving functional outcomes and quality of life. Randomized controlled trials have shown that neurofeedback, when added to standard rehabilitation, leads to significant gains in self-efficacy, cognitive insight, and attention, as well as increased serum levels of neuroplasticity markers such as brain-derived neurotrophic factor (BDNF) and reelin, which are associated with enhanced neuroplasticity and functional recovery (Markiewicz et al., 2021a, 2021b, 2024a, 2024b). Targeted neurofeedback approaches, including EEG and real-time fMRI protocols, have demonstrated the ability to modulate specific neural circuits implicated in schizophrenia, such as the superior temporal gyrus and frontoparietal networks. This modulation is associated with reductions in treatment-resistant auditory hallucinations and improvements in working memory, self-referential processing, and social functioning (Kobayashi et al., 2025). Meta-analytic evidence further supports the potential of neurofeedback to improve emotion regulation, anxiety, and depressive symptoms in psychiatric populations, with medium to large effect sizes, although heterogeneity remains high and further multicenter trials are needed (Pindi et al., 2022). Further research is essential to optimize NFB protocols, validate its long-term benefits, and explore its integration into standard care for schizophrenia.

This systematic review aims to critically analyze the existing evidence on the efficacy of NFB in these types of disorders. The goal is to assess the innovative potential of this technique, identifying its strengths, limitations, and future perspectives for the treatment of patients with schizophrenia or other schizophrenia spectrum disorders.

## Methods

### Search Strategies

**Fig. 1:**
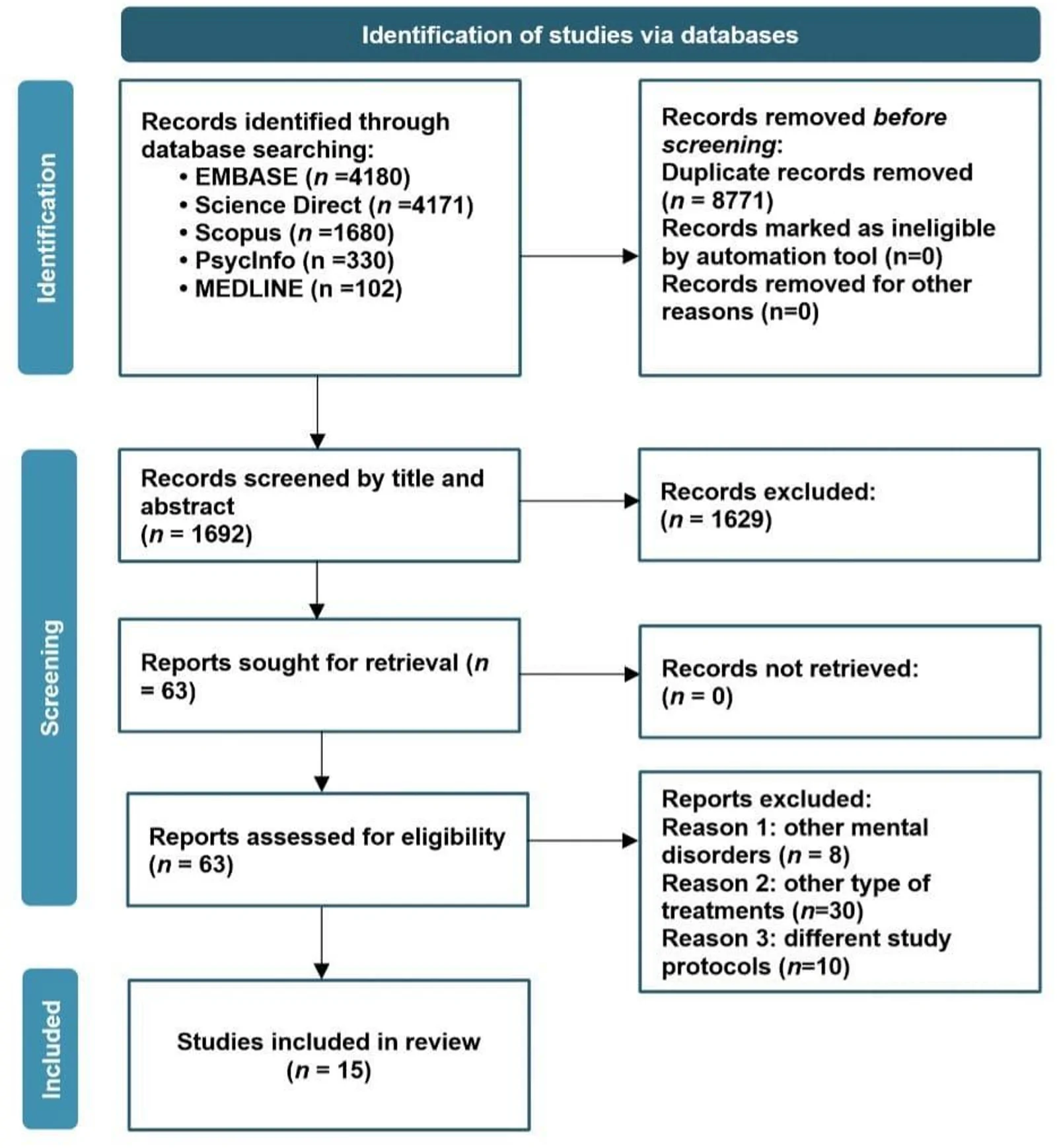
PRISMA. Flow diagram 2020

The systematic review was fully conducted according to PRISMA guidelines 2020 for Systematic Reviews by PRISMA Group (Page et al., 2021) (Fig. 1)*.* From September 2024 until November 2024), the researchers G.C.P, S.T., G.S. and S.L. searched the databases EMBASE, ScienceDirect, Scopus, PsycInfo and MEDLINE/Pubmed for relevant studies using the following search terms string: (“eeg-biofeedback” OR “eeg-BFB” OR “neurofeedback”) AND (“psychosis OR schizophrenia”) AND (“neurocognitive changes”) AND (“humoral changes”) AND (cognitive training). The electronic searching was supplemented by hand-searching reference lists of the included review articles to identify any additional sources. The recorded protocol is available on OSF (https://doi.org/10.17605/OSF.IO/AME3Z).

### Eligibility Criteria

We included every article written in the English language without temporal restrictions concerning publication date, meeting the following criteria:

- *Participants*: patients with Schizophrenia Spectrum Disorders, Psychosis or with Clinical High Risk for Psychosis.
- *Intervention*: any kind of Neurofeedback based intervention.
- *Comparison*: therapy as usual (TAU) or control groups without Neurofeedback intervention; healthy control groups undergoing the same treatment of the experimental group with psychotic subjects; comparison of pre- and post-intervention in single-group studies.
- *Outcomes*: We considered the outcomes of neurocognitive and humoral changes.
- *Study design*: clinical trials that include repeated measures to allow for both pre- and post-intervention comparisons in the experimental group, as well as comparisons between the experimental and control groups.

### Exclusion Criteria

We excluded every article written in languages other than English meeting the following criteria:

- *Participants*: studies involving patients with disorders other than schizophrenia spectrum disorders (e.g., mood disorders, anxiety disorders, or personality disorders).
- *Intervention*: studies that do not includeNeurofeedback-based interventions or that use biofeedback techniques not based on EEG.
- *Comparison*: studies that do not use “treatment as usual” (TAU) as a comparison group.
- *Outcomes*: studies that do not assess neurocognitive or humoral changes as primary outcomes.
- *Study design*: articles that are not clinical trials (e.g., reviews, meta-analyses, preclinical or animal studies) or studies with significant methodological flaws (e.g., lack of a control group, inadequate sample size, or other major biases).

### Data Extraction

The researchers G.C.P, S.T., G.S. and S.L. extracted data using a format which included: characteristics of studies and samples, type of intervention, target symptoms to be treated, frequency and duration of the interventions, outcomes and their assessments, follow-up if present (Table 1).

**Table 1:** Data extraction

Authors	Year	Title	Nation	Study design	Sample	Population	Measures	Interventions	Outcome
Cordes, J. S., Mathiak, K. A., Dyck, M., Alawi, E. M., Gaber, T. J., Zepf, F. D., Klasen, M., Zvyagintsev, M., Gur, R. C., & Mathiak, K.	2015	Cognitive and neural strategies during control of the anterior cingulate cortex by fMRI neurofeedback in patients with schizophrenia.	Germany	RCT	*N* = 22	11 patients with schizophrenia and 11 healthy controls	PANAS; QMI; PANSS	NF training based on real-time functional magnetic resonance imaging (fMRI)	Schizophrenia patients activated the dorsal ACC subsection during fMRI NF training, while controls activated the rostral subsection; Different cognitive strategies were reported: music-related in schizophrenia patients, sports-related in healthy controls; The difference in strategies did not contribute to the difference in neural activation; Patients achieved functional recovery in ACC signal regulation through compensation from less impaired domains (cognitive processing, musical imagination); Social reward feedback (smiling avatar) was used, potentially improving conditioning and long-term transfer in patients; Results suggest the need for specific neural and cognitive targets to normalize dysfunctions in schizophrenia patients.
Choi, J., Corcoran, C. M., Fiszdon, J. M., Stevens, M., Javitt, D. C., Deasy, M., Haber, L. C., Dewberry, M. J., & Pearlson, G. D.	2017	Pupillometer-based neurofeedback cognitive training to improve processing speed and social functioning in individuals at clinical high risk for psychosis	USA	Double blind RCT	*N* = 62	Clinical high risk for psychosis	WAIS-III Digit Symbol-Coding subtest (primary outcome for processing speed); MCT, secondary measure for processing speed; CPT-IP, for sustained attention/vigilance; WAIS-III WMI; SAS-SR, primary measure for social functioning); SAS-A, secondary measure for social functioning); SIPS/SOPS; BDI-II	PST; ACT	PST group showed significant improvements in processing speed at post-treatment and 2-month follow-up compared to active control; PST group demonstrated higher overall social adjustment at 2-month follow-up; Improvements in Coding subtest were associated with better overall social adjustment and less avoidance/distress in new social situations; Low attrition rate in PST group (10%) suggests good engagement with the intervention; Neurofeedback based PST improved processing speed and social functioning in individuals at clinical high risk for psychosis.
Kirschner, M.	2017	Self-regulation of the dopaminergic reward system via real-time fMRI neurofeedback in schizophrenia	Switzerland	Clinical trial	*N* = 28	14 patients with schizophrenia and 14 healthy controls	fMRI activation of SN/VT; Neurofeedback learning performance; Self-reported negative symptoms (scale not specified)	Real-time fMRI neurofeedback targeting SN/VTA; abstract visual feedback of midbrain dopaminergic activity; positive mental imagery (recalling rewarding scenes) to up-regulate SN/VTA activation.	Schizophrenia patients were unable to actively self-regulate SN/VTA activity and failed to improve self-regulation across rtfMRI-NF sessions, unlike healthy controls who showed successful up-regulation. Impaired neurofeedback learning in patients was significantly associated with higher levels of negative symptoms, particularly diminished expression.
Orlov, N. D., Giampietro, V., O’Daly, O., Lam, S. L., Barker, G. J., Rubia, K., McGuire, P., Shergill, S. S., Allen, P.	2018	Real-time fMRI neurofeedback to down-regulate superior temporal gyrus activity in patients with schizophrenia and auditory hallucinations: a proof-of-concept study.	UK	Clinical trial	*N* = 12	Schizophrenia	PANSS; PsyRats; WASI	fMRI; rtfMRI-NF	Patients with schizophrenia and AVH learned to down-regulate activity in the left STG through rtfMRI neurofeedback; Increased functional connectivity between frontal and temporal language regions; Patients maintained the ability to reduce STG activity even without visual feedback (transfer scan); Changes in functional connectivity were associated with changes in AVH symptom severity.
Rieger, K., Rarra, M. H., Diaz Hernandez, L., Hubl, D., Koenig, T.	2018	Neurofeedback-Based Enhancement of Single-Trial Auditory Evoked Potentials: Treatment of Auditory Verbal Hallucinations in Schizophrenia.	Switzerland	RCT	*N* = 10	Schizophrenia	EEG; PANSS; PsyRat; HVLR-R; NAB-Mazes; D2 test of attention; HCS.	N100 ERP modulation NF	No significant pre/post intervention differences in AVH symptoms assed with HCS.A positive association between learning factors and improvements in hallucinations. No relation between psychotic symptoms and NF performance. No significant pre/post intervention spatial difference or GFP effect on N100. A significant spatial pre/post difference for P200(control group) with a tendency for higher GFP post-training.
Balconi, M., Frezza, A., Vanutelli, M. E.	2018	Emotion Regulation in Schizophrenia: A Pilot Clinical Intervention as Assessed by EEG and Optical Imaging (Functional Near-Infrared Spectroscopy).	Italy	RCT	*N* = 18	Schizophrenia	fNIRS; EEG	Emotional Neurofeedback training	Patients preserved the ability to assess emotional valence of pictures, but had difficulty determining arousal levels; Initial assessment showed prevalence of Delta waves in patients’ cortical activity during emotional processing; Neurofeedback training restored a balanced interhemispheric state, as evidenced by fNIRS (O2Hb levels) and EEG (frontal Delta LTA) measurements; After training, patients rated negative stimuli more positively compared to initial assessment; The integration of fNIRS and EEG proved effective in evaluating the efficacy of neurofeedback treatment by elucidating cortical oscillation and hemodynamic effects in the prefrontal cortex.
Balconi and Vannutelli	2019	Neurofeedback Intervention for Emotional Behavior Regulation in Schizophrenia: New Experimental Evidences from Optical Imaging	Italy	RCT	*N* = 25	Schizophrenia	fNIRS; SAM.	NF	After the intervention, the perception of negative and positive stimuli received a more positive value compared to the initial assessment. Increase in oxygenated hemoglobin over frontopolar regions for positive and negative stimuli compared to neutral ones.
Choi, J., Fiszdon, J., Stevens, M., Haber, L., & Pearlson, G	2019	11.2 PUPILLOMETER-BASED NEUROFEEDBACK COGNITIVE TRAINING: OPTIMIZING TASK ENGAGEMENT TO ENHANCE LEARNING IN PRODROME, FIRST EPISODE, AND ESTABLISHED PSYCHOSIS	USA	Double blind RCT	*N* = 100	Prodrome, First episode, and established psychosis	Processing speed, motivation/interest, and treatment completion.	CT with pupillometric neurofeedback: Training with the pupillometer actively adjusting task difficulty based on physiological feedback; CT without neurofeedback: Training with the pupillometer turned off; progression based on correct/incorrect responses only.	Greater improvement in motor and non-motor processing speed in neurofeedback groups; Better adherence: 90% completed training with neurofeedback versus 72% without; Optimized cognitive load using pupillometric feedback, allowing for personalized task adjustments, ensuring optimal engagement without overloading cognitive resources.
Zweerings, J., Hummel, B., Keller, M., Zvyagintsev, M., Schneider, F., Klasen, M., Mathiak, K.	2019	Neurofeedback of core language network nodes modulates connectivity with the default-mode network: A double-blind fMRI neurofeedback study on auditory verbal hallucinations	Germany	Double blind RCT	*N* = 56		Resting-state functional connectivity Well-being assessment (four weeks after training)	Two days of rtfMRI neurofeedback training targeting the left-hemispheric language network, including the IFG and pSTG. Participants learned to down- and up-regulate their brain activation in these regions.	Increased coupling between language and DMN nodes after down-regulation compared to up-regulation neurofeedback; More pronounced increases in functional connectivity between language network and DMN nodes in patients compared to healthy individuals; Down-regulation led to increased coupling between language network nodes and bilateral IPL and PCC/precuneus in patients; Up-regulation strengthened connectivity with the mPFC. Improved well-being four weeks post-training predicted increased functional coupling between left IFG and left IPL; RtfMRI neurofeedback may modulate brain network function relevant to auditory verbal hallucinations in schizophrenia.
Singh, F., Shu, I. W., Hsu, S. H., Link, P., Pineda, J. A., & Granholm, E.	2020	Modulation of frontal gamma oscillations improves working memory in schizophrenia	USA	Clinical trial	*N* = 31	Schizophrenia.	EEG neurofeedback for gamma band activity, n-back working memory task, neuropsychological assessments (processing speed, reasoning)	EEG neurofeedback sessions targeting frontal gamma oscillations.	Improved working memory, processing speed, reasoning, problem-solving, and reduced psychiatric symptoms.
R. Tamano; T. Ogawa; C. Cai; M. Kawanabe	2020	P.413 Towards using EEG-Neurofeedback for the treatment of psychotic symptoms and working memory in schizophrenia: a feasibility study in healthy subjects	Japan	Clinical trial	*N* = 11	Schizophrenia.	EEG data; fMRI data; SPQ; N-back test for working memory; WLS score	EEG-neurofeedback training:	High AUC value (0.76) in predicting disease-related label from EEG in a specific subject; Mean AUC value of 0.65 across subjects; No significant decrease in WLS and SPQ after NFB training; Significant increase in d-prime for 4-back test after NFB; High correlation between change in 3-back test d-prime and change in WLS.
Markiewicz., Markiewicz-Gospodarek, A., Dobrowolska, B., Łoza, B.	2021	Improving Clinical, Cognitive, and Psychosocial Dysfunctions in Patients with Schizophrenia: A Neurofeedback Randomized Control Trial.	Poland	RCT	*N*= 44	Schizophrenia.	Clinical (PANSS), cognitive (CTT, d2), psychosocial (BCIS, AIS, and GSES), and electrophysiological parameters (QEEG_NF) BDNF levels	Neurofeedback/rehabilitation (NF); Standard rehabilitation (R) programs	NF training significantly improved BDNF levels, self-efficacy (GSES), and clinical symptoms in schizophrenia patients compared to standard rehabilitationNF also enhanced cognitive functions (working memory, concentration) and psychosocial outcomes (illness acceptance, reflectiveness)
Markiewicz, R., Markiewicz-Gospodarek, A., Trubalski, M., Łoza, B.	2024	Neurocognitive, Clinical and Reelin Activity in Rehabilitation Using Neurofeedback Therapy in Patients with Schizophrenia	Poland	RCT	*N* = 37	Schizophrenia.	d2; BCIS; AIS; GSES; CTT; PANSS; Serum reelin levels measured using ELISA.	NF	This study found that neurofeedback therapy, when added to antipsychotic treatment, led to a significant increase in the serum reelin level in schizophrenia patients. Neurofeedback also improved neurocognitive functions and reduced negative and general symptoms of schizophrenia (measured by PANSS scores). These results align with previous studies showing that cognitive and social skills training enhances neuroplasticity, particularly in brain regions involved in problem-solving and emotional processing. However, this study emphasized symptom changes alongside cognitive improvements, suggesting a broader benefit of neurofeedback therapy compared to traditional cognitive rehabilitation approaches.
Morfini, F., Bauer, C. C. C., Zhang, J., Whitfield-Gabrieli, S., Shinn, A. K., Niznikiewicz, M. A.	2024	Targeting the superior temporal gyrus with real-time fMRI neurofeedback: A pilot study of the indirect effects on self-referential processes in schizophrenia	USA	Randomized, participant-blinded, sham-controlled pilot study	*N* = 22	Schizophrenia	fMRI neurofeedback targeting the superior temporal gyrus, self-referential processing task, fMRI activation analysis.	One session of real-time fMRI neurofeedback (STG or motor cortex)	Increased activation in the medial prefrontal cortex, ACC, and superior frontal cortex during self-referential processing.
Dhanis, H., Gninenko, N., Morgenroth, E., Potheegadoo, J., Rognini, G., Faivre, N., Blanke, O., & Van De Ville, D.	2024	Real-time fMRI neurofeedback modulates induced hallucinations and underlying brain mechanisms	Switzerland	Clinical trial, Pilot study	*N* = 20	Clinical high risk for psychosis	fMRI neurofeedback on fronto-parietal network, robot-induced hallucination task, neural activity tracking.	Three days of fMRI neurofeedback to control hallucination-related brain networks.	Improved control over hallucination sensitivity, with sustained neural network changes indicating potential therapeutic implications.

ACC = Anterior Cingulate Cortex; AIS = Acceptance of Illness Scale; BCIS = Beck Cognitive Insight Scale; BDI-II = Beck Depression Inventory—Second Edition; BDNF = Brain-Derived Neurotrophic Factor; CT = Cognitive Training; CTT = Color Trails Test; CPT-IP = Continuous Performance Test—Identical Pairs; D2 test = D2 Test of Attention; DMN = Default Mode Network; EEG = Electroencephalogram; ELISA = Enzyme-Linked Immunosorbent Assay; fMRI = Functional Magnetic Resonance Imaging; fNIRS = Functional Near-Infrared Spectroscopy; GSES = General Self-Efficacy Scale; HCS = Hallucination Change Scale; HVLR-R= Hopkins Verbal Learning Test-Revised; IFG = Inferior Frontal Gyrus; IPL = Inferior Parietal Lobe; mPFC = Medial Prefrontal Cortex; MCT = Minnesota Clerical Test; NAB-Mazes = Neuropsychological Assessment Battery—Mazes subtest; PANAS = Positive and Negative Affect Schedule; PANSS = Positive and Negative Syndrome Scale; PCC = Posterior Cingulate Cortex; pSTG = Posterior Superior Temporal Gyrus; PsyRats = Psychotic Rating Symptom Scale; QEEG_NF = Quantitative EEG Neurofeedback; QMI = Betts’ Questionnaire upon Mental Imagery; SAM = Self-Assessment Manikin; SAS-A = Social Anxiety Scale for Adolescents; SAS-SR = Social Adjustment Scale—Self Report; SIPS/SOPS = Structured Interview for Prodromal Syndromes / Scale of Prodromal Symptoms; SPQ = Schizotypal Personality Questionnaire; STG = Superior Temporal Gyrus; WAIS-III = Wechsler Adult Intelligence Scale—Third Edition; WASI = Wechsler Abbreviated Scale of Intelligence; WLS = Weighted Linear Summation; WMI = Working Memory Index

The articles that we agreed upon were included in the next screening process. However, in cases where there was no agreement between the evaluators, a brief discussion was held, and a final decision was made jointly by the team on whether to include the articles in the next step.

### Risk of Bias Assessment

**Fig. 2:**
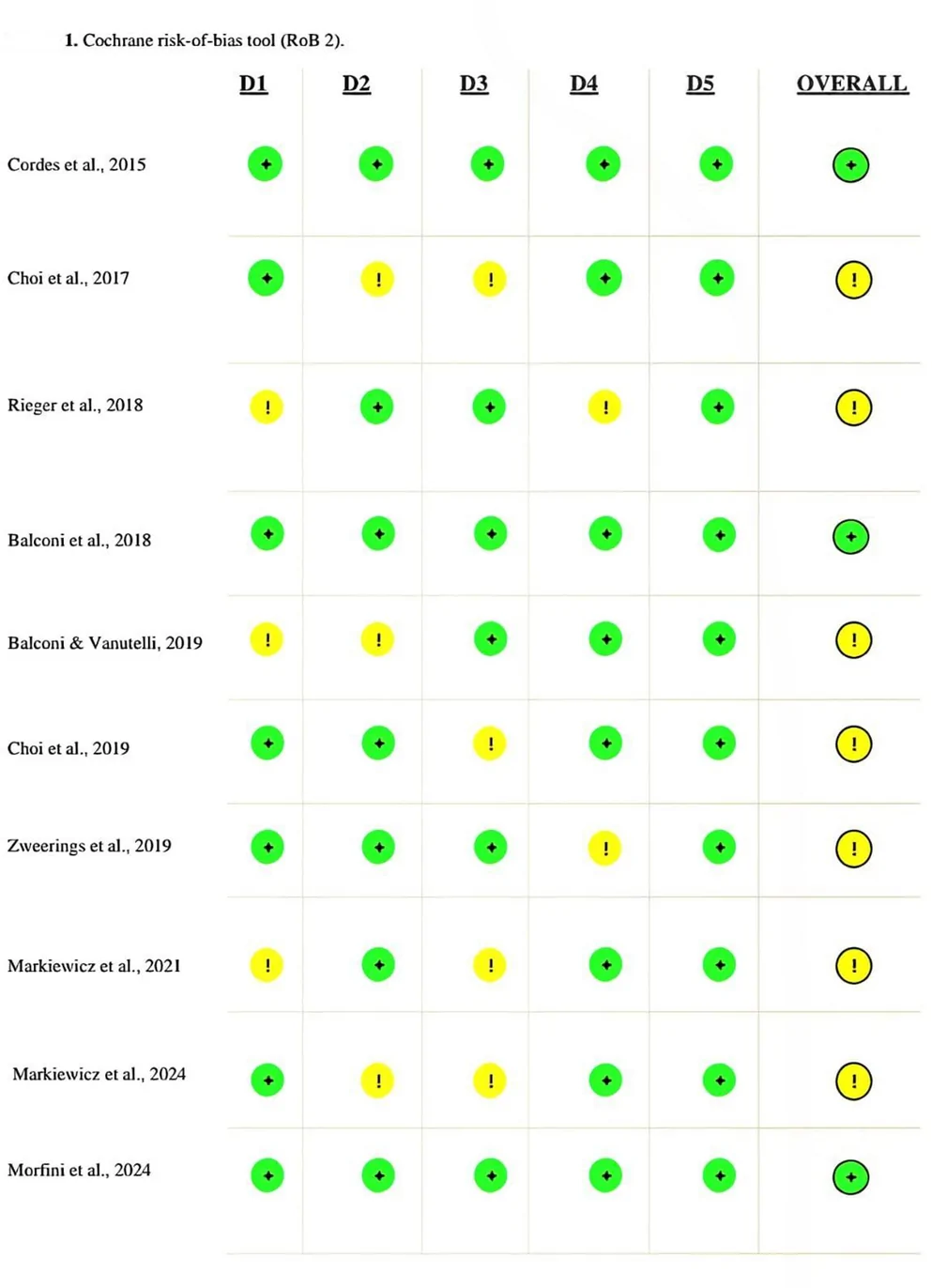
Cochrane risk-of-bias tool for randomized trials (RoB 2)

**Fig. 3:**
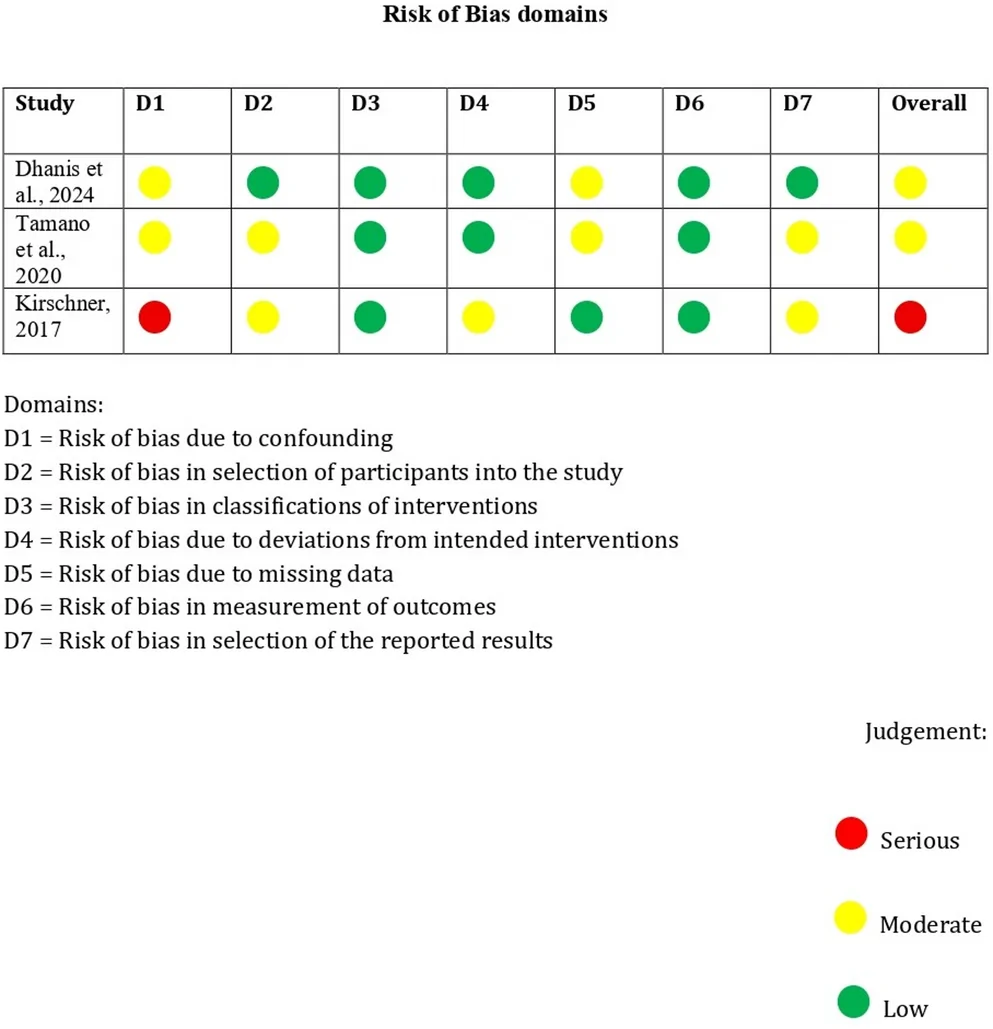
Cochrane risk-of-bias tool for non-randomized - studies of Interventions (ROBINS-I)

**Fig. 4:**
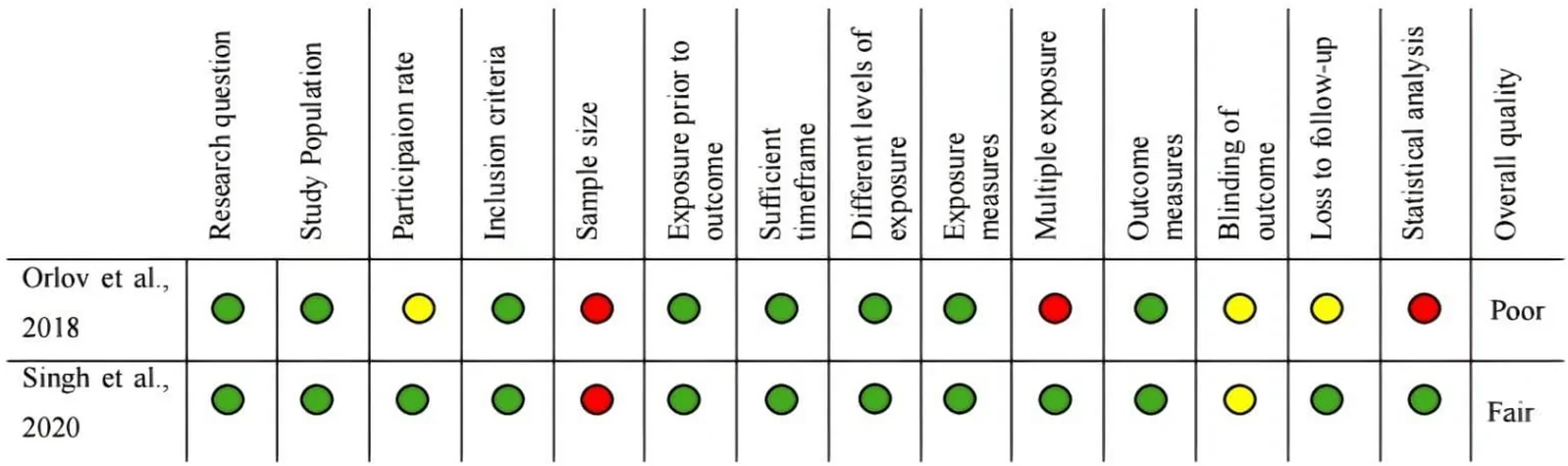
NIH quality assessment tool

The risk of bias for the included studies was assessed with the Cochrane risk-of-bias tool for randomized trials, version 2 (RoB 2) by Sterne et al. (2019) (Fig. 2). Regarding other non-randomized studies, we used ROBINS-I (Sterne et al., 2016) (Fig. 3); NIH Quality Assessment Tool for observational studies or single-group pilots (NIH, 2024) (Fig. 4).

## Results

### Study Selection

The database search yielded 10,463 reports. After removing duplicates, 1692 unique reports remained. Following title and abstract screening, 1617 records were excluded based on relevance. Of the 63 studies assessed for eligibility, 48 were excluded due to inclusion of other mental disorders (*n* = 8), use of techniques other than neurofeedback (*n* = 30), or inappropriate study design (*n* = 10). Consequently, 15 studies meeting eligibility criteria were included in this systematic review, as illustrated in the PRISMA flowchart.

### Characteristics of Included Studies

#### Cognitive and Social-Emotional Functioning

Cordes et al. (2015) investigated functional MRI-based neurofeedback targeting the anterior cingulate cortex in schizophrenia patients. Patients demonstrated limited but significant ability to regulate ACC activity, though less consistently than healthy controls. Greater success in ACC regulation correlated with improvements in negative symptoms and cognitive functioning. Despite these promising results, practical barriers including the high cost and limited accessibility of fMRI technology pose challenges for widespread implementation.

Balconi et al. (2018, 2019) conducted studies using EEG and functional Near-Infrared Spectroscopy to evaluate emotional neurofeedback training. Results revealed significant improvements in the experimental groups, with increased prefrontal activity and more balanced neural activation patterns. Participants demonstrated a positive shift in ratings for both negative and positive emotional stimuli post-training, with increased oxygenated hemoglobin in frontopolar regions. These findings suggest enhanced implicit emotion regulation through adaptive prefrontal activation, even when explicit emotional recognition remained unchanged.

These studies collectively demonstrate that neurofeedback targeting cognitive control and emotional regulation networks can produce measurable improvements in processing speed, social functioning, and emotional processing in schizophrenia spectrum disorders. The convergence of findings across different neurofeedback modalities (fMRI, EEG, fNIRS, pupillometry) suggests robust effects on prefrontal cortex function. However, variability in patient response highlights the importance of individualized approaches, and the challenge remains to translate these laboratory gains into accessible clinical interventions given current technological and cost barriers. Studies in CHR individuals are discussed separately due to their distinct clinical status.

#### Auditory Hallucinations and Superior Temporal Gyrus Modulation

Orlov et al. (2018) evaluated real-time fMRI neurofeedback targeting the superior temporal gyrus in twelve patients with persistent auditory hallucinations. The neurofeedback group successfully reduced STG activity during sessions, leading to significant reductions in severity, frequency, and distress associated with auditory hallucinations. These improvements persisted one week post-intervention and extended to broader psychotic symptoms including delusions. The intervention was well-tolerated with no adverse effects reported.

Rieger et al. (2018) explored EEG-based neurofeedback targeting the auditory N100 event-related potential in ten patients with auditory verbal hallucinations. While no significant group-level improvements in hallucination-specific symptoms were observed, better learning during N100 training sessions correlated with greater reductions in auditory hallucinations in individual participants. Interestingly, changes in the P200 component were observed across all participants, suggesting broader neurofeedback effects beyond the specific training protocol.

Morfini et al. (2024) conducted a randomized trial with twenty-two adults comparing STG-targeted versus motor cortex-targeted neurofeedback. The STG experimental group exhibited significantly greater post-neurofeedback activation in anterior regions of the self-reference network, including the medial prefrontal cortex, anterior cingulate cortex, and superior frontal cortex. These findings suggest that modulating STG activity influences broader self-referential networks relevant to hallucination psychopathology.

The evidence across these studies indicates that neurofeedback targeting the STG can successfully reduce activity in this hallucination-associated region and produce clinically meaningful symptom improvements. The persistence of benefits beyond the intervention period is particularly encouraging. However, the variability in outcomes, particularly in the EEG study, suggests that success may depend on individual learning capacity and that optimal protocols require further refinement. The mechanistic link between STG modulation and changes in self-referential processing networks provides important insights into how neurofeedback may address the neural underpinnings of auditory hallucinations.

#### Brain Network Connectivity

Zweerings et al. (2019) investigated resting-state connectivity changes following real-time fMRI neurofeedback in twenty-one schizophrenia patients and thirty-five healthy individuals. Participants underwent two days of neurofeedback training targeting left hemispheric language network regions (inferior frontal gyrus and posterior superior temporal gyrus). Patients exhibited significantly greater functional connectivity increases between language network and default mode network nodes compared to healthy controls. Downregulation neurofeedback led to enhanced connectivity between language network nodes and bilateral inferior parietal lobe and posterior cingulate cortex in patients, while upregulation strengthened connectivity with medial prefrontal cortex. Improved well-being four weeks post-training predicted increased functional coupling between left IFG and left IPL.

This study demonstrates that neurofeedback can induce lasting changes in large-scale brain network organization relevant to auditory hallucinations in schizophrenia. The differential effects of upregulation versus downregulation protocols on specific network connections suggest that targeted modulation strategies can be designed based on desired connectivity outcomes. The persistence of connectivity changes and their correlation with clinical improvements four weeks post-training provides compelling evidence for neurofeedback’s capacity to produce durable neuroplastic changes that may underlie symptom reduction.

#### Working Memory and Gamma Oscillations

Singh et al. (2020) investigated EEG neurofeedback training to enhance frontal gamma-band response in thirty-one schizophrenia patients. Participants completed twelve weeks of twice-weekly gamma-neurofeedback training sessions. Cognitive improvements were demonstrated in n-back task performance and MATRICS Consensus Cognitive Battery tests. Significant enhancement in 1-back performance was observed at four, eight, and twelve weeks, while substantial 2-back improvements emerged by week twelve. The total MCCB score showed significant improvement across all time points, with particularly marked advancements in speed of processing, working memory, and reasoning/problem-solving domains by week twelve. While not the primary treatment target, significant changes were also observed in general psychopathology and PANSS subscales.

This study provides strong evidence that targeted modulation of gamma oscillations can produce dose-dependent improvements in working memory and broader cognitive functions in schizophrenia. The progressive nature of improvements, with more challenging tasks requiring longer training duration, suggests that neurofeedback effects accumulate over time and that treatment protocols should be calibrated to cognitive task difficulty. The additional benefits for general psychopathology, though secondary, indicate potential broader therapeutic effects beyond the primary cognitive targets.

#### Findings in CHR Individuals

Two studies specifically investigated neurofeedback interventions in individuals at Clinical High Risk for psychosis. Choi et al. (2017, 2019) applied pupillometer-based neurofeedback to enhance processing speed and task engagement. CHR participants showed improved processing speed and social functioning, with higher treatment adherence when pupillometry-guided difficulty adjustment was used. These findings highlight that neurofeedback may exert beneficial cognitive effects even before the onset of full psychosis, although they should not be generalized to schizophrenia populations due to substantial differences in clinical profiles, symptom expression, and developmental trajectories.

#### Multidimensional Rehabilitation Approaches

Markiewicz et al. (2021a, 2021b, 2024a, 2024b) conducted two studies investigating neurofeedback as adjunctive therapy integrated with comprehensive rehabilitation programs. The 2021 study with forty-four male patients demonstrated that neurofeedback combined with standard rehabilitation produced superior gains in neurocognitive and psychosocial functioning compared to standard rehabilitation alone, accompanied by greater increases in BDNF levels. The 2024 study with thirty-seven male patients showed significant improvements in clinical symptoms and neurocognitive functioning in the neurofeedback group, with serum reelin levels positively correlating with symptom improvement. Both studies integrated neurofeedback with daily rehabilitation activities including social training, cognitive exercises, and art therapy.

These studies demonstrate that neurofeedback can be effectively integrated into comprehensive rehabilitation programs for schizophrenia, producing benefits that extend beyond those achieved with standard rehabilitation alone. The elevation of neuroplasticity biomarkers (BDNF and reelin) alongside clinical improvements provides biological evidence for neurofeedback’s mechanisms of action. The structured, multimodal approach combining neurofeedback with psychosocial interventions may represent an optimal treatment model that addresses multiple dimensions of schizophrenia pathology simultaneously.

#### Novel Mechanisms and Therapeutic Strategies

Dhanis et al. (2024) employed real-time fMRI neurofeedback combined with MR-compatible robotics to train participants in modulating brain networks associated with presence hallucinations. Over three training days, participants successfully upregulated a specific “PH-network,” resulting in heightened sensitivity to presence hallucination experiences. Neural adaptations included increased PH-network activity during hallucination-inducing conditions and reduced activation during control conditions, changes that correlated with successful training modulation.

This innovative study demonstrates that neurofeedback can be used not only to reduce pathological brain activity but also to systematically modulate specific network dynamics to better understand hallucination mechanisms. The ability to induce controlled changes in hallucination-related networks offers both scientific insights into the neural basis of these experiences and potential therapeutic applications for managing hallucinations in neuropsychiatric conditions. This bidirectional modulation approach represents a promising avenue for developing targeted network-based interventions.

## Discussion

The findings from this systematic review emphasize the promising role of EEG-biofeedback (or neurofeedback) as a potential adjunctive therapy for schizophrenia spectrum disorders and psychosis. The reviewed studies collectively demonstrate that neurofeedback interventions have shown promise in improving cognitive functions (Enriquez-Geppert et al., 2014; Surmeli et al., 2016), emotional regulation, and certain biochemical markers such as brain-derived neurotrophic factor (BDNF) and serum reelin levels (Markiewicz et al., 2021a, 2021b, 2024a, 2024b). By targeting both neurocognitive impairments and biochemical changes, neurofeedback interventions offer a unique therapeutic avenue for addressing persistent symptoms often resistant to conventional treatments. However, despite encouraging preliminary results, several aspects require further exploration to establish clinical utility.

### Efficacy and Mechanisms of Action

Neurofeedback demonstrates the capacity to modulate brain activity in key regions implicated in auditory hallucinations and emotional dysregulation. For example, Orlov et al. (2018) showed that neurofeedback targeting the superior temporal gyrus (STG) significantly reduced STG activity, which corresponded with a decrease in the severity and distress of auditory hallucinations. These effects were sustained even after the intervention, suggesting a durable therapeutic benefit. Additionally, studies such as Zweerings et al. (2019) observed enhanced functional connectivity between the language network and the default mode network (DMN), highlighting neurofeedback’s role in promoting neural reorganization. These findings indicate a dual mechanism: symptom management and underlying neural reorganization: in fact, neurofeedback not only targets symptoms but also promotes neuroplasticity (Enriquez-Geppert et al., 2019). Importantly, this modulation may offer a novel approach to mitigating persistent symptoms that are resistant to conventional treatments.

However, the exact pathways through which neurofeedback induces these effects remain poorly understood. While improvements in cognitive functions, such as working memory and emotional regulation, are evident, the variability in outcomes, particularly for patients with severe baseline impairments—suggests that neurocognitive reserve may influence response to treatment (Hammond, 2011). Moreover, heterogeneity in neurofeedback protocols, target outcomes, and intervention durations complicates cross-study comparisons. Future studies should aim to standardize methodologies and explore biomarkers, such as BDNF and reelin, to elucidate the mechanisms underlying neurofeedback-induced neuroplasticity.

Importantly, findings from CHR individuals were discussed separately, as CHR populations differ substantially from patients with schizophrenia or established psychosis in terms of symptom severity, risk trajectories, and functional impairment. Therefore, results observed in CHR samples, particularly those related to processing speed and early cognitive changes, should not be generalized to schizophrenia populations. This distinction is essential to avoid overinterpretation and to maintain conceptual clarity between preventive interventions and clinical treatment studies.

### Clinical and Biochemical Outcomes

The clinical benefits observed, including improved processing speed, attention, and social functioning, are particularly relevant given their direct impact on patients’ quality of life. For example, Choi et al. (2017, 2019) reported improved processing speed and social functioning in high-risk individuals and those experiencing first-episode psychosis following pupillometer-based neurofeedback training. These findings are particularly relevant, as enhanced cognitive performance is directly associated with better daily functioning and quality of life in patients with schizophrenia spectrum disorders(Kober et al., 2015).

On a biochemical level, neurofeedback interventions were associated with increased level of brain derived neurotrophic factor (BDNF) and serum reelin, as reported by Markiewicz et al. (2021a, 2021b, 2024a, 2024b). These markers provide a compelling biological rationale for neurofeedback’s role in cognitive rehabilitation. However, the clinical significance of these findings remains uncertain. For example, while improved functional connectivity and biochemical markers are promising, their translation to long-term symptom relief and real-world functioning has yet to be conclusively demonstrated (Birbaumer et al., 2013). Additionally, the small sample size and limited diversity of populations in many studies restrict the generalizability of results. Longer follow-up periods are needed to determine whether neurofeedback-induced changes are durable and clinically meaningful. A more systematic approach to patient selection and evaluation could address these shortcomings.

### Challenges and Future Directions

A major challenge identified in the current literature is the lack of standardization across NFB protocols. Substantial variability exists in methodologies, including the use of EEG- versus fMRI-based NFB, differences in targeted brain regions, EEG frequency bands, training duration, and feedback modalities, making cross-study comparison and synthesis difficult. For instance, while some studies primarily targeted symptom-specific outcomes such as auditory hallucinations (Orlov et al., 2018), others were oriented toward broader cognitive domains, including working memory and attentional functioning (Singh et al., 2020). Establishing standardized protocols is therefore essential to improve replicability, support meta-analytic work, and ultimately contribute to evidence-based clinical guidelines. Advancing toward universally accepted methodological standards will require coordinated efforts and collaboration between multidisciplinary research and clinical teams. Another important challenge concerns the integration of NFB within existing treatment frameworks. Although NFB has emerged as a promising adjunctive intervention, its comparative and additive effects relative to established treatments, such as antipsychotic medications and cognitive-behavioral therapies, remain inadequately explored (Strehl, 2014). Moreover, patient-specific factors including motivation, cognitive reserve, symptom severity, and adherence may significantly influence training outcomes. Tailoring NFB protocols to these individual characteristics and exploring hybrid treatment approaches (e.g., NFB combined with personalized psychotherapy modules) could potentially enhance therapeutic efficacy (Thibault et al., 2018). A further limitation lies in the incomplete and non-standardized reporting of demographic and clinical variables across the included studies. Because this review adopts a descriptive and narrative approach, the heterogeneity and frequent absence of such data limited their integration into the synthesis. Insufficient reporting reduces the ability to evaluate these factors as potential confounders and restricts the generalizability of the findings. Future research would benefit from more systematic reporting of demographic, clinical, and treatment-related characteristics, allowing for more precise interpretation of their contribution to treatment response. Future investigations should prioritize large-scale, longitudinal RCTs employing standardized methodologies and extended follow-up periods. Such trials should evaluate not only the immediate effects of NFB, but also its long-term impact on clinical symptoms, neurocognitive functioning, daily functioning, and quality of life. Further, the development of personalized NFB protocols grounded in individual neurocognitive or neurophysiological profiles holds promise for improving effectiveness. Parallel research on cost-effectiveness, accessibility, and feasibility will also be crucial for determining the practicality of integrating NFB into routine clinical practice. In addition, more efforts should be directed toward elucidating the mechanisms underlying EEG-based NFB. Combining EEG-NFB with neuroimaging modalities such as fMRI could help clarify the neural pathways modulated by training. For example, Morfini et al. (2024) demonstrated that real-time fMRI NFB targeting the superior temporal gyrus not only reduced auditory hallucinations but also modulated networks involved in self-referential processing, offering valuable insight into cognitive mechanisms in schizophrenia. Such multimodal approaches may assist in identifying biomarkers of treatment response and guiding the development of more targeted and mechanistically informed interventions. Lastly, integrating NFB with emerging therapeutic technologies, such as virtual-reality-based interventions or transcranial magnetic stimulation, may open new avenues for multimodal, comprehensive treatment strategies. Strengthening interdisciplinary collaboration will be key to accelerating progress in this evolving field. A further promising yet underexplored area for future research concerns humoral biomarkers. Only a limited number of studies have investigated changes in inflammatory, neurotrophic, or hormonal markers following NFB interventions, despite mounting evidence that such biomarkers play a critical role in the pathophysiology of psychosis and other psychiatric disorders. Expanding research in this domain could help clarify whether NFB induces measurable biological changes beyond neural modulation and whether these changes mediate cognitive or clinical improvements. Moreover, identifying humoral markers associated with treatment response may support the development of more personalized NFB protocols and contribute to a deeper mechanistic understanding of how NFB interacts with broader psychophysiological systems. Given the increasing recognition of immunological and endocrine dysregulation in psychosis, incorporating humoral measures into future NFB trials represents a valuable and timely direction for the field.

## Conclusion

This systematic review highlights the potential of neurofeedback as a promising adjunctive therapy for schizophrenia spectrum disorders and psychosis. The reviewed studies suggest that neurofeedback interventions can enhance cognitive functions, improve emotional regulation, and influence biochemical markers associated with neuroplasticity. By targeting both neurocognitive impairments and neural reorganization, neurofeedback presents a unique therapeutic avenue for addressing symptoms that are often resistant to conventional treatments. Future research should prioritize large-scale, randomized controlled trials with extended follow-up periods to assess both the efficacy and durability of neurofeedback-induced changes.

## Data Availability

No datasets were generated or analysed during the current study.

## References

[CR1] American Psychiatric Association (2022). *Diagnostic and statistical manual of mental disorders* (5th ed.). American Psychiatric Publishing.

[CR2] Askovic, M., Soh, N., Elhindi, J., & Harris, A. W. F. (2023). Neurofeedback for post-traumatic stress disorder: Systematic review and meta-analysis of clinical and neurophysiological outcomes. *European Journal of Psychotraumatology*, *14*(2), 2257435. 10.1080/20008066.2023.2257435 PMC10515677 37732560

[CR3] Balconi, M., Frezza, A., & Vanutelli, M. E. (2018). Emotion regulation in schizophrenia: A pilot clinical intervention as assessed by EEG and optical imaging (functional near-infrared spectroscopy). *Frontiers in Human Neuroscience*, *12*, 395. 10.3389/fnhum.2018.00395 PMC6189325 30356708

[CR4] Balconi, M., & Vanutelli, M. E. (2019). Neurofeedback intervention for emotional behavior regulation in schizophrenia: New experimental evidences from optical imaging. *NeuroRegulation*, *6*(2). 10.15540/NR.6.2.71

[CR5] Birbaumer, N., Ruiz, S., & Sitaram, R. (2013). Learned regulation of brain metabolism. *Trends in Cognitive Sciences*, *17*(6), 295–302. 10.1016/j.tics.2013.04.009 23664452

[CR6] Charlson, F. J., Ferrari, A. J., Santomauro, D. F., Diminic, S., Stockings, E., Scott, J. G., McGrath, J. J., & Whiteford, H. A. (2018). Global epidemiology and burden of schizophrenia: Findings from the global burden of disease study 2016. *Schizophrenia Bulletin*, *44*(6), 1195–1203. 10.1093/schbul/sby058 PMC6192504 29762765

[CR7] Chiasson, P., Boylan, M. R., Elhamiasl, M., Pruitt, J. M., Ranjan, S., Riels, K., Sahoo, A. K., Mirifar, A., & Keil, A. (2023). Effects of neurofeedback training on performance in laboratory tasks: A systematic review. *International Journal of Psychophysiology: Official Journal of the International Organization of Psychophysiology*, *189*, 42–56. 10.1016/j.ijpsycho.2023.04.005 37148977

[CR8] Choi, J., Corcoran, C. M., Fiszdon, J. M., Stevens, M., Javitt, D. C., Deasy, M., Haber, L. C., Dewberry, M. J., & Pearlson, G. D. (2017). Pupillometer-based neurofeedback cognitive training to improve processing speed and social functioning in individuals at clinical high risk for psychosis. *Psychiatric Rehabilitation Journal*, *40*(1), 33–42. 10.1037/prj0000217 PMC5326611 27560455

[CR9] Choi, J., Fiszdon, J., Stevens, M., Haber, L., & Pearlson, G. (2019). 11.2 Pupillometer-based neurofeedback cognitive training: Optimizing task engagement to enhance learning in prodrome, first episode, and established psychosis. *Schizophrenia Bulletin*, *45*(2), 104–105. 10.1093/schbul/sbz022.040

[CR10] Cordes, J. S., Mathiak, K. A., Dyck, M., Alawi, E. M., Gaber, T. J., Zepf, F. D., Klasen, M., Zvyagintsev, M., Gur, R. C., & Mathiak, K. (2015). Cognitive and neural strategies during control of the anterior cingulate cortex by fMRI neurofeedback in patients with schizophrenia. *Frontiers in Behavioral Neuroscience*, *9*, 169. 10.3389/fnbeh.2015.00169 PMC4480149 26161073

[CR11] Dhanis, H., Gninenko, N., Morgenroth, E., et al. (2024). Real-time fMRI neurofeedback modulates induced hallucinations and underlying brain mechanisms. *Commun Biol*, *7*, 1120. 10.1038/s42003-024-06842-x PMC11391061 39261559

[CR12] Enriquez-Geppert, S., Huster, R. J., & Herrmann, C. S. (2014). EEG neurofeedback as a tool to modulate cognition and behavior: A review tutorial. *Frontiers in Human Neuroscience*, *8*, 547. 10.3389/fnhum.2014.00547 PMC5319996 28275344

[CR50] Enriquez-Geppert, S., Smit, D., Pimenta, M. G., & Arns, M. (2019). Neurofeedback as a treatment intervention in ADHD: Current evidence and practice. *Current Psychiatry Reports, 21*(6), 46. 10.1007/s11920-019-1021-4 PMC6538574 31139966

[CR13] Gandara, V., Pineda, J. A., Shu, I. W., & Singh, F. (2020). A systematic review of the potential use of neurofeedback in patients with schizophrenia. *Schizophrenia Bulletin Open*, *1*(1), sgaa005. 10.1093/schizbullopen/sgaa005 PMC7418870 32803157

[CR14] Gruzelier, J. H. (2014). EEG-neurofeedback for optimising performance. I: A review of cognitive and affective outcome in healthy participants. *Neuroscience and Biobehavioral Reviews*, *44*, 124–141. 10.1016/j.neubiorev.2013.09.015 24125857

[CR15] Hammond, D. C. (2011). What is neurofeedback: An update. *Journal of Neurotherapy*, *15*(4), 305–336. 10.1080/10874208.2011.623090

[CR16] Kobayashi, Y., Asai, T., Yoshihara, Y., Yamashita, M., Nakamura, H., Shimizu, M., Kawashima, T., Miyata, J., Kawato, M., Murai, T., Imamizu, H., & Takahashi, H. (2025). Enhancement of the left frontoparietal network through real-time functional magnetic resonance imaging functional connectivity-informed neurofeedback and its impact on working memory in schizophrenia: A pilot study. *Psychiatry and Clinical Neurosciences*, *79*(9), 531–544. 10.1111/pcn.13849 PMC12405832 40545821

[CR17] Kober, S. E., Witte, M., Ninaus, M., Neuper, C., & Wood, G. (2015). Learning to modulate one’s own brain activity: The effect of spontaneous mental strategies. *Frontiers in Human Neuroscience*, *9*, 295. 10.3389/fnhum.2015.00295 PMC3798979 24151462

[CR18] Leichsenring, F., Steinert, C., & Ioannidis, J. P. A. (2019). Toward a paradigm shift in treatment and research of mental disorders. *Psychological Medicine*, *49*(13), 2111–2117. 10.1017/S0033291719002265 31474241

[CR19] Li, Z., Ren, H., Tian, Y., Zhou, J., Chen, W., OuYang, G., Chen, Z., Yi, W., Song, H., Xie, P., Wang, X., Chen, X., Xiao, Q., & Liu, H. (2024). Neurofeedback technique for treating male schizophrenia patients with impulsive behavior: A randomized controlled study. *Frontiers in Psychiatry*, *15*, 1472671. 10.3389/fpsyt.2024.1472671 PMC11491364 39435128

[CR20] Lutgens, D., Gariepy, G., & Malla, A. (2017). Psychological and psychosocial interventions for negative symptoms in psychosis: Systematic review and meta-analysis. *The British Journal of Psychiatry: the Journal of Mental Science*, *210*(5), 324–332. 10.1192/bjp.bp.116.197103 28302699

[CR21] Markiewicz, R., Dobrowolska, B., & Wójcik, M. (2021a). The impact of neurofeedback training on BDNF serum level in patients with schizophrenia: A pilot study. *Frontiers in Psychiatry*, *12*, 645. 10.3389/fpsyt.2021.645

[CR23] Markiewicz, R., Markiewicz-Gospodarek, A., Dobrowolska, B., & Łoza, B. (2021b). Improving clinical, cognitive, and psychosocial dysfunctions in patients with schizophrenia: A neurofeedback randomized control trial. *Neural Plasticity*, *2021*, 4488664. 10.1155/2021/4488664 PMC8380506 34434228

[CR22] Markiewicz, R., Dobrowolska, B., & Wójcik, M. (2024a). Neurofeedback training increases serum reelin levels in patients with schizophrenia: A randomized controlled trial. *Schizophrenia Research*, *238*, 150–157. 10.1016/j.schres.2024.01.010

[CR24] Markiewicz, R., Markiewicz-Gospodarek, A., Trubalski, M., & Łoza, B. (2024b). Neurocognitive, clinical and reelin activity in rehabilitation using neurofeedback therapy in patients with schizophrenia. *Journal of Clinical Medicine*, *13*(14), 4035. 10.3390/jcm13144035 PMC11277514 39064075

[CR25] Marzbani, H., Marateb, H. R., & Mansourian, M. (2016). Neurofeedback: A comprehensive review on system design, methodology and clinical applications. *Basic and Clinical Neuroscience*, *7*(2), 143–158. 10.15412/J.BCN.03070208 PMC4892319 27303609

[CR26] Morfini, F., Bauer, C. C. C., Zhang, J., Whitfield-Gabrieli, S., Shinn, A. K., & Niznikiewicz, M. A. (2024). Targeting the superior temporal gyrus with real-time fMRI neurofeedback: A pilot study of the indirect effects on self-referential processes in schizophrenia. *Schizophrenia Research*, *270*, 358–365. 10.1016/j.schres.2024.06.036 PMC11531859 38968807

[CR27] National Institutes of Health (NIH). *Study Quality Assessment Tools*. Retrieved 07 November, 2024 from https://www.nhlbi.nih.gov/health-topics/study-quality-assessment-tools

[CR28] Oprea, D. C., Mawas, I., Moroșan, C. A., Iacob, V. T., Cămănaru, E. M., Cristofor, A. C., Dobrin, R. P., Gireadă, B., Petrariu, F. D., & Chiriță, R. (2024). A systematic review of the effects of EEG neurofeedback on patients with schizophrenia. *Journal of Personalized Medicine*, *14*(7), 763. 10.3390/jpm14070763 PMC11278179 39064017

[CR29] Orlov, N. D., Giampietro, V., O’Daly, O., Lam, S. L., Barker, G. J., Rubia, K., McGuire, P., Shergill, S. S., & Allen, P. (2018). Real-time fMRI neurofeedback to down-regulate superior temporal gyrus activity in patients with schizophrenia and auditory hallucinations: A proof-of-concept study. *Translational Psychiatry*, *8*(1), 46. 10.1038/s41398-017-0067-5 PMC5865171 29430009

[CR30] Orndorff-Plunkett, F., Singh, F., Aragón, O. R., & Pineda, J. A. (2017). Assessing the effectiveness of neurofeedback training in the context of clinical and social neuroscience. *Brain Sciences*, *7*(8), 95. 10.3390/brainsci7080095 PMC5575615 28783134

[CR31] Page, M. J., McKenzie, J. E., Bossuyt, P. M., Boutron, I., Hoffmann, T. C., Mulrow, C. D., et al. (2021). The PRISMA 2020 statement: An updated guideline for reporting systematic reviews. *Bmj*, *372*, n71. 10.1136/bmj.n71es PMC8005924 33782057

[CR32] Pazooki, K., Leibetseder, M., Renner, W., et al. (2019). Neurofeedback treatment of negative symptoms in schizophrenia: Two case reports. *Applied Psychophysiology and Biofeedback*, *44*, 31–39. 10.1007/s10484-018-9417-1 PMC6373527 30267339

[CR33] Pindi, P., Houenou, J., Piguet, C., & Favre, P. (2022). Real-time fMRI neurofeedback as a new treatment for psychiatric disorders: A meta-analysis. *Progress in Neuro-Psychopharmacology & Biological Psychiatry*, *119*, 110605. 10.1016/j.pnpbp.2022.110605 35843369

[CR35] Rieger, K., Rarra, M. H., Diaz Hernandez, L., Hubl, D., & Koenig, T. (2018). Neurofeedback-based enhancement of single-trial auditory evoked potentials: Treatment of auditory verbal hallucinations in schizophrenia. *Clinical EEG and Neuroscience*, *49*(6), 367–378. 10.1177/1550059418765810 29569473

[CR36] Singh, F., Shu, I. W., Hsu, S. H., Link, P., Pineda, J. A., & Granholm, E. (2020). Modulation of frontal gamma oscillations improves working memory in schizophrenia. *NeuroImage Clinical*, *27*, 102339. 10.1016/j.nicl.2020.102339 PMC7390812 32712452

[CR37] Solmi, M., Seitidis, G., Mavridis, D., Correll, C. U., Dragioti, E., Guimond, S., Tuominen, L., Dargél, A., Carvalho, A. F., Fornaro, M., Maes, M., Monaco, F., Song, M., Shin, I., J., & Cortese, S. (2023). Incidence, prevalence, and global burden of schizophrenia—data, with critical appraisal, from the global burden of disease (GBD) 2019. *Molecular Psychiatry*, *28*(12), 5319–5327. 10.1038/s41380-023-02138-4 37500825

[CR38] Sterne, J. A. C., Hernán, M. A., Reeves, B. C., et al. (2016). ROBINS-I: A tool for assessing risk of bias in non-randomised studies of interventions. *BMJ*, *355*, i4919. 10.1136/bmj.i4919 PMC5062054 27733354

[CR39] Sterne, J. A. C., Savović, J., Page, M. J., Elbers, R. G., Blencowe, N. S., Boutron, I., Cates, C. J., Cheng, H. Y., Corbett, M. S., Eldridge, S. M., et al. (2019). RoB 2: A revised tool for assessing risk of bias in randomised trials. *Bmj*, *366*, l4898. 10.1136/bmj.l4898 31462531

[CR40] Strehl, U. (2014). What learning theories can teach us in designing neurofeedback treatments. *Frontiers in Human Neuroscience*, *8*, 894. 10.3389/fnhum.2014.00894 PMC4222234 25414659

[CR41] Surmeli, T., Ertem, A., Eralp, E., & Kos, I. H. (2016). Schizophrenia and the efficacy of qEEG-guided neurofeedback treatment: A clinical case series. *Clinical EEG and Neuroscience*, *47*(2), 133–144. 10.1177/1550059415613592 22715481

[CR42] Thibault, R. T., Lifshitz, M., & Raz, A. (2018). The self-regulating brain and neurofeedback: Experimental science and clinical promise. *Cortex; A Journal Devoted To the Study of the Nervous System and Behavior*, *106*, 86–100. 10.1016/j.cortex.2017.12.024 26706052

[CR43] Turiaco, F., Iannuzzo, F., Genovese, G., Lombardo, C., Silvestri, M. C., Celebre, L., Muscatello, M. R. A., & Bruno, A. (2024). Cognitive effects of brief and intensive neurofeedback treatment in schizophrenia: A single center pilot study. *AIMS Neuroscience*, *11*(3), 341–351. 10.3934/Neuroscience.2024021 PMC11486612 39431270

[CR44] Wahbeh, M. H., & Avramopoulos, D. (2021). Gene-environment interactions in schizophrenia: A literature review. *Genes*, *12*(12), 1850. 10.3390/genes12121850 PMC8702084 34946799

[CR45] Zhao, Z., Li, X., Feng, G., Shen, Z., Li, S., Xu, Y., Huang, M., & Xu, D. (2018). Altered effective connectivity in the default network of the brains of first-episode, Drug-Naïve schizophrenia patients with auditory verbal hallucinations. *Frontiers in Human Neuroscience*, *12*, 456. 10.3389/fnhum.2018.00456 PMC6289978 30568584

[CR46] Zweerings, J., Hummel, B., Keller, M., Zvyagintsev, M., Schneider, F., Klasen, M., & Mathiak, K. (2019). Neurofeedback of core language network nodes modulates connectivity with the default-mode network: A double-blind fMRI neurofeedback study on auditory verbal hallucinations. *Neuroimage*, *189*, 533–542. 10.1016/j.neuroimage.2019.01.058 30703519

